# Post-cholecystectomy Choleperitoneum Due to Cystic Duct Stump Leak: A Case Report Emphasizing Diagnostic Challenges and Multidisciplinary Management

**DOI:** 10.7759/cureus.102983

**Published:** 2026-02-04

**Authors:** Archit Garg, Mehak Bassi, Lara Calegari, Aadhithyaraman Santharaman, Andrew Korman, Sugirdhana Velpari, Arkady Broder

**Affiliations:** 1 Internal Medicine, Saint Peter's University Hospital, Rutgers Robert Wood Johnson Medical School, New Brunswick, USA; 2 Gastroenterology and Hepatology, TidalHealth Peninsula Regional, Salisbury, USA; 3 Gastroenterology and Hepatology, Saint Peter's University Hospital, Rutgers Robert Wood Johnson Medical School, New Brunswick, USA

**Keywords:** bile duct injury, biloma, cholecystectomy, choleperitoneum, common bile duct

## Abstract

Biliary peritonitis, also known as choleperitoneum, is a rare but severe complication of cholecystectomy. The symptoms are non-specific, which can cause a delay in the diagnosis, leading to potential sepsis and multi-organ failure. A 45-year-old female underwent robotic cholecystectomy for gallstone pancreatitis and cholecystitis. One week post-operatively, she presented again with abdominal pain and distention, rapidly progressing to septic shock with respiratory, renal, and neurological failure. Initial imaging showed only mild ascites. Imaging showed a biliary leak, and paracentesis confirmed a biliary peritonitis by revealing bilious fluid (ascitic bilirubin 25 mg/dL). The patient underwent endoscopic retrograde cholangiopancreatography (ERCP) with biliary stent placement and was started on antibiotics, with improvement in clinical condition. The case highlights the diagnostic difficulty of choleperitoneum, where early imaging can be subtle. Timely, multidisciplinary management involving peritoneal drainage, ERCP with stent placement, and prophylactic antibiotics is necessary; otherwise, biliary peritonitis is associated with significant mortality and morbidity. This report underscores the need for vigilance even after technically uneventful surgery, particularly in high-risk patients.

## Introduction

Cholecystectomy remains one of the most commonly performed intra-abdominal surgeries, with more than 750,000 operations performed annually in the United States [1]. However, its potential for significant morbidity remains, primarily through iatrogenic bile duct injury. While the incidence of bile duct injury is low (0.1-0.2% in open cholecystectomy and 0.3-0.8% through laparoscopic technique [2]), the subsequent leakage of bile into the peritoneal cavity leading to choleperitoneum constitutes a surgical emergency [3]. Bile is a potent chemical irritant and can cause chemical peritonitis by direct tissue injury and by inciting an inflammatory cascade (recruitment of neutrophils and monocytes through cellular immune activation via cytokines, chemokines, and adhesion molecules), leading to vasodilation, fluid sequestration, and bacterial superinfection [4]. This can precipitate rapid clinical deterioration into septic shock and multi-organ failure [3].

The diagnostic challenge of choleperitoneum is its insidious onset and non-specific early presentation. Symptoms such as diffuse abdominal pain, anorexia, or malaise are easily attributed to normal postoperative convalescence. This leads to a delay in recognition. Moreover, with the rapid integration of robotic-assisted platforms into cholecystectomy practice (increasing 37-fold between 2010 and 2019), a new dimension has been introduced to the complication landscape [5]. Despite enhanced visualization and instrument articulation, robotic cholecystectomy in comparison to laparoscopy is associated with higher rates of bile duct injury requiring operative repair (0.7% vs. 0.2%) and biliary interventions (7.4% vs. 6.0%) [5]. When combined with anatomical challenges such as severe inflammation, anomalous tracts, or high BMI, these robotic platforms may yield unique clinical scenarios despite precision [6].

Despite extensive documentation on choleperitoneum, data on extremely rapid progression from vague symptoms to life-threatening multi-organ failure following a minimally invasive approach are limited [7,8]. Most bile collections evolve insidiously over weeks, and symptoms may be discounted initially (in a study by Lee et al., symptoms occurred after an average of 16.8 days, and symptoms were dismissed in 77% of cases) [7,9]. We present a case of post-cholecystectomy choleperitoneum that vividly illustrates this diagnostic odyssey. Our patient, with multiple risk factors including a high BMI and significant biliary inflammation, underwent robotic-assisted cholecystectomy and developed a cystic duct stump leak. She presented with non-specific symptoms that escalated to septic shock with multi-organ failure.

This report highlights the diagnostic pitfalls encountered and emphasizes the importance of a systematic, timely investigative protocol and the role of a coordinated, multidisciplinary management strategy. Moreover, these case reports fill a critical gap by demonstrating that even with advanced surgical technology, bile-related complications can manifest with unexpected severity, reinforcing the need for sustained postoperative vigilance.

## Case presentation

A 45-year-old female with a past medical history of hypertension presented to the emergency department with moderate, non-radiating epigastric pain, nausea, and one episode of non-bloody, non-bilious vomiting. Physical examination was significant for high BMI (47 kg/m^2^) and epigastric and right upper quadrant tenderness. Laboratory investigations showed mild leukocytosis (13.5 x 10^3^/mm^3^), transaminitis (aspartate aminotransferase (AST) 253 U/L, aspartate aminotransferase (ALT) 206 U/L, alkaline phosphatase (ALP) 80 U/L, and total bilirubin 0.9 mg/dL and elevated lipase 9071 U/L). Computed tomography (CT) abdomen revealed peripancreatic fat stranding and a distended gallbladder. Right upper quadrant ultrasound revealed cholelithiasis with gallbladder distention and mild pericholecystic fluid. These findings were consistent with acute pancreatitis secondary to possible gallstones (given the absence of alcohol consumption) with concomitant acute cholecystitis. Her pancreatitis was managed conservatively, and after 36 hours of conservative management, she underwent a robotic-assisted cholecystectomy for her cholecystitis. No external gallbladder drainage was performed. The patient had biochemical and clinical improvement, and she was discharged two days after surgery.

Seven days later, she returned with worsening abdominal pain, decreased appetite, nausea, and respiratory distress. Physical examination was significant for abdominal tenderness in all quadrants. The patient was tachycardic (heart rate 102 beats/minute) and hypoxemic on room air (SpO_2_ 88%). Laboratory examination was positive for leukocytosis (28 x 103/mm^3^), slight transaminitis (AST 57 U/L, ALT 96 U/L), elevated ALP (246 U/L), and total bilirubin 2.1 mg/dL, deranged kidney function tests (blood urea nitrogen (BUN) 47 mg/dL and Cr 5.83 mg/dL), and acidosis (venous pH 7.21). Pertinent lab values are mentioned in Table 1. CT abdomen and ultrasound indicated new mild ascites and post-cholecystectomy changes. The patient was admitted to the intensive care unit with the working diagnosis of severe sepsis from an abdominal source leading to septic shock and multi-organ failure (including acute respiratory failure, toxic metabolic encephalopathy, and renal failure). The patient was started on continuous bilevel positive airway pressure (BiPAP), antibiotics (piperacillin and tazobactam), continuous hydration, and pain control. Owing to patients' clinical instability, further testing could not be done immediately. Her condition kept deteriorating, with the patient requiring hemodialysis due to persistent oliguria and worsening kidney function (uptrending creatinine, hyperkalemia, hyperphosphatemia, and acidosis). She also developed ileus and required nasogastric tube placement.

**Table 1:** Pertinent Lab Values

Pertinent Tests	Patient Value	Normal Reference Range
White blood cell count (WBC)	28 × 10^3^/mm^3^	4.0-10.0 × 10^3^/mm^3^
Aspartate aminotransferase (AST)	57 U/L	10-40 U/L
Alanine aminotransferase (ALT)	96 U/L	7-56 U/L
Alkaline phosphatase (ALP)	246 U/L	44-147 U/L
Total bilirubin	2.1 mg/dL	0.1-1.2 mg/dL
Blood urea nitrogen (BUN)	47 mg/dL	7-20 mg/dL
Creatinine (Cr)	5.83 mg/dL	0.6-1.3 mg/dL
Venous pH	7.21	7.31-7.41

**Figure 1:**
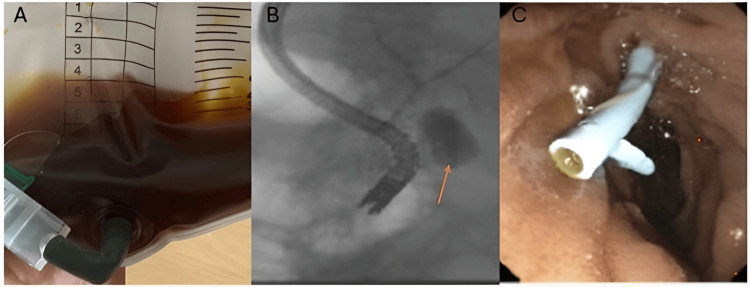
Biliary Drainage and Endoscopic Findings A: This depicts the output from the biliary drain, which is dark yellowish-brown in color, suggestive of bile. B: The arrow depicts the leakage of bile during a cholangiogram performed when the ERCP was done. C: The image depicts the biliary stent placed during the ERCP. ERCP: endoscopic retrograde cholangiopancreatography

After 36 hours of aggressive supportive care (including initiation of hemodialysis), her condition stabilized sufficiently to allow for further definitive diagnostic testing. On the second day of hospitalization, magnetic resonance imaging (MRI)/magnetic resonance cholangiopancreatography (MRCP) showed worsening of ascites and non-visualization of a segment of the common bile duct just distal to the right and left biliary ductal confluence, suggestive of bile duct injury. This was confirmed by a hepatobiliary iminodiacetic acid (HIDA) scan, which showed a biliary leak. Paracentesis showed brown colored ascitic fluid with elevated bilirubin (25 mg/dL), indicating choleperitoneum. A percutaneous drain placed by interventional radiology immediately drained bilious fluid (Figure 1A). Subsequently, endoscopic retrograde cholangiopancreatography (ERCP) was performed on the third day of hospitalization. The cholangiogram during ERCP revealed extravasation of contrast from the cystic duct stump, confirming the site of the bile leak (Figure 1B). In response, a 10 Fr x 7 cm plastic stent was placed in the common bile duct to facilitate drainage and promote closure of the leak (Figure 1C). The repeat HIDA scan showed no leak. However, the patient had continued leukocytosis, and a repeat CT scan showed persistent perihepatic fluid collection on the fifth day of hospitalization. Hence, a drain was placed in the perihepatic region. The cultures from this region grew *Candida albicans* and *Streptococcus,* for which she required a long course of antibiotics. The patient was started on fluconazole, and piperacillin-tazobactam was switched to ampicillin. Her liver enzymes and leukocyte count decreased over the next few days, and the biliary drain was removed. The patient’s kidney function and respiratory status improved, so hemodialysis and BiPAP were discontinued. The patient was discharged on a long course of antibiotics for two weeks with the hepatic drain in place. On subsequent follow-up in one week, the hepatic drain was also removed.

## Discussion

Biliary peritonitis or choleperitoneum refers to severe inflammation resulting from contact of the bile with the peritoneal surface. This may occur due to bile duct injury during cholecystectomy. Every year, around 400 bile duct injury cases are reported in the US, with the rates being twice that of laparoscopic cholecystectomy as compared to open cholecystectomy [10]. Clinically significant bile duct leaks develop in 0.7-1.4% laparoscopic cholecystectomy cases [11]. Moreover, a recent study has shown that despite technical advantages in terms of enhanced visualization and instrumentation, robotic surgery does not eliminate the risk of biliary injury. In fact, it was shown to be associated with higher rates of bile duct injury (0.4% vs. 0.2%, relative risk (RR) 1.88) requiring more operative repair (0.7% vs. 0.2%) and biliary interventions (7.4% vs. 6.0%) [5]. Biliary duct injury during cholecystectomy can be due to inadvertent partial or complete bile duct transection, crushing by clamps, thermal energy from electrocautery, or ischemic injury from tight occlusion with clips, causing biliary leak [12]. Other causes include slippage of cystic duct ligature, leakage from anomalous bile ducts, or functional hypertension in the biliary system due to ongoing inflammation and elevated liver function tests (LFTs), causing biliary leak even from a minor trauma to the small biliary ducts during cholecystectomy [5]. Major bile duct injuries, although less common, are one of the most dreaded complications due to the associated technical complexity in management and potential for long-term morbidity. It is important to note that many injuries, whether major or subtle, may not be recognized intra-operatively and instead present in the postoperative period, as illustrated in our case.

Risk factors that predispose to biliary peritonitis post cholecystectomy include age over 40 years, preoperative abnormal LFTs, subacute inflammation of the gallbladder, cholecystolithiasis complicated with effusion, gallbladder wall thickening, laparoscopic surgery, inexperienced surgeon, poor anatomy, and excess inflammation around the gallbladder region [13]. In our case, the patient had five risk factors mentioned above (age >40 years old, abnormal LFTs, subacute gallbladder inflammation, cholelithiasis with effusion, and laparoscopic intervention). Studies suggest that less than 25% cases of bile duct injury are detected during the cholecystectomy or during that particular hospitalization, and the majority of the patients present later due to symptoms from continuous bile leakage, which can later cause peritonitis [11]. The symptoms develop within a week of surgery, but sometimes can linger and get worse even after two to three weeks after surgery [13]. The presentation can range from diffuse abdominal pain, anorexia, nausea, vomiting, fatigue, to abdominal distension, fever, chills, jaundice, ascites, ileus, and ultimately multi-organ failure [11,12]. In our case, the patient presented after one week of surgery with abdominal distension, pain, and nausea.

Radionuclide imaging (HIDA scan) can be done to visualize the leakage of bile from the duct into the peritoneal cavity [14]. CT abdomen and ultrasound are also done to detect primary pathology and peri-hepatic collections [14]. However, the definitive diagnosis is made by analysis of fluid from paracentesis. The ascitic fluid, which appears dark orange or brown in color and fluid analysis, shows bilirubin more than the serum bilirubin with an absolute value of >6 mg/dL (normal ascitic bilirubin is 0.7-0.8 mg/dL), along with a normal ascitic fluid amylase level [15-17]. Studies have shown that ascitic fluid bilirubin of >6 mg/dL and an ascitic fluid/serum bilirubin ratio of >3.25-5 is sensitive and specific for choleperitoneum [15-17]. In the current case, the HIDA scan showed a biliary leak, with CT abdomen and ultrasound showing ascites and peri-hepatic collections. The paracentesis showed brown colored fluid with a bilirubin concentration of 25 mg/dL and an ascitic fluid/serum bilirubin ratio of 11.9, confirming the diagnosis of choleperitoneum.

The mainstay in the management of choleperitoneum involves surgical intervention. Collections over 4 cm in diameter do not resolve without intervention and get complicated with secondary bacterial peritonitis, cholangitis, sepsis, and multi-organ failure [11,17]. In our case, it got complicated with infectious peritonitis, sepsis, and multi-organ failure involving the kidneys, lungs, and the brain, requiring ICU level of care. Hence, surgical intervention with peritoneal toilet and lavage was done. Additionally, prophylactic antibiotic therapy and biliary drainage were done by placing an external drain to remove the bile in the abdomen, which can be a source of infection, causing complicated superimposed bacterial peritonitis [18]. Therefore, timely diagnosis and management of biliary peritonitis is imperative, as biliary peritonitis following a cholecystectomy serves as an independent indicator of postoperative mortality [18]. In this case, placement of a peritoneal drain to remove the bile, along with antibiotic therapy and biliary stent placement to address the leak, resulted in a favorable recovery without long-term complications.

## Conclusions

This case demonstrates the fulminant trajectory of choleperitoneum following robotic cholecystectomy in a patient with high-risk features like acute cholecystitis, obesity, and concurrent pancreatitis, which increase bile duct injury risk intra-operatively. Despite robotic technology’s sophistication, they carry a higher rate of bile duct injury requiring operative repair compared to laparoscopy, making it important for clinicians to maintain a high index of suspicion in these patients for subtle signs of bile duct injury, as they can progress to multiorgan failure. Furthermore, this case reinforces that technological advancement does not eliminate classic surgical pitfalls and underscores the imperative for heightened postoperative vigilance, low threshold for advanced imaging, and prompt multidisciplinary intervention in high-risk populations.

## References

[1] Stinton LM, Shaffer EA (2012). Epidemiology of gallbladder disease: cholelithiasis and cancer. Gut Liver.

[2] Karvonen J, Gullichsen R, Laine S, Salminen P, Grönroos JM (2007). Bile duct injuries during laparoscopic cholecystectomy: primary and long-term results from a single institution. Surg Endosc.

[3] Pekolj J, Alvarez FA, Palavecino M, Sánchez Clariá R, Mazza O, de Santibañes E (2013). Intraoperative management and repair of bile duct injuries sustained during 10,123 laparoscopic cholecystectomies in a high-volume referral center. J Am Coll Surg.

[4] Allen K, Jaeschke H, Copple BL (2011). Bile acids induce inflammatory genes in hepatocytes: a novel mechanism of inflammation during obstructive cholestasis. Am J Pathol.

[5] Kalata S, Thumma JR, Norton EC, Dimick JB, Sheetz KH (2023). Comparative safety of robotic-assisted vs laparoscopic cholecystectomy. JAMA Surg.

[6] Wang J, Johnson NW, Casey L (2023). Robotic colon surgery in obese patients: a systematic review and meta-analysis. ANZ J Surg.

[7] Lee CM, Stewart L, Way LW (2000). Postcholecystectomy abdominal bile collections. Arch Surg.

[8] Woldehana NA, Jung A, Parker BC, Coker AM, Haut ER, Adrales GL (2025). Clinical outcomes of laparoscopic vs robotic-assisted cholecystectomy in acute care surgery. JAMA Surg.

[9] Fletcher DR, Hobbs MS, Tan P (1999). Complications of cholecystectomy: risks of the laparoscopic approach and protective effects of operative cholangiography: a population-based study. Ann Surg.

[10] Connor S, Garden OJ (2006). Bile duct injury in the era of laparoscopic cholecystectomy. Br J Surg.

[11] Schofer JM (2010). Biliary causes of postcholecystectomy syndrome. J Emerg Med.

[12] Yang S, Hu S, Gu X, Zhang X (2022). Analysis of risk factors for bile duct injury in laparoscopic cholecystectomy in China: a systematic review and meta-analysis. Medicine (Baltimore).

[13] Paladugu R, Rau A, Schein M, Wise L (1998). Spontaneous perforation of the hepatic duct in adults. Dig Surg.

[14] Runyon BA (1987). Ascitic fluid bilirubin concentration as a key to choleperitoneum. J Clin Gastroenterol.

[15] Darwin P, Goldberg E, Uradomo L (2010). Jackson Pratt drain fluid-to-serum bilirubin concentration ratio for the diagnosis of bile leaks. Gastrointest Endosc.

[16] DeBenedet AT, Scheiman JM, Elta GH, Elmunzer BJ (2013). Peritoneal fluid bilirubin to serum bilirubin ratio for the diagnosis of bile leaks in orthotopic liver transplant recipients. Dig Dis Sci.

[17] Daldoul S, Moussi A, Zaouche A (2012). T-tube drainage of the common bile duct choleperitoneum: etiology and management. J Visc Surg.

[18] Díaz-Martínez J, Chapa-Azuela O, Roldan-García JA, Flores-Rangel GA (2020). Bile duct injuries after cholecystectomy, analysis of constant risk. Ann Hepatobiliary Pancreat Surg.

